# Biochemical Assessment of Coenzyme Q_10_ Deficiency

**DOI:** 10.3390/jcm6030027

**Published:** 2017-03-05

**Authors:** Juan Carlos Rodríguez-Aguilera, Ana Belén Cortés, Daniel J. M. Fernández-Ayala, Plácido Navas

**Affiliations:** 1 Laboratorio de Fisiopatología Celular y Bioenergética, 41013 Sevilla, Spain; jcrodagu@upo.es (J.C.R.-A.); abcorrod@upo.es (A.B.C.); 2 Centro de Investigación Biomédica en Red de Enfermedades Raras, Instituto de Salud Carlos III, Universidad Pablo de Olavide-CISC, 41013 Sevilla, Spain; dmorfer@upo.es; 3 Centro Andaluz de Biología del Desarrollo, 41013 Sevilla, Spain

**Keywords:** coenzyme Q_10_, CoQ_10_ deficiency syndrome, CoQ_10_ biosynthesis, mitochondria diseases

## Abstract

Coenzyme Q_10_ (CoQ_10_) deficiency syndrome includes clinically heterogeneous mitochondrial diseases that show a variety of severe and debilitating symptoms. A multiprotein complex encoded by nuclear genes carries out CoQ_10_ biosynthesis. Mutations in any of these genes are responsible for the primary CoQ_10_ deficiency, but there are also different conditions that induce secondary CoQ_10_ deficiency including mitochondrial DNA (mtDNA) depletion and mutations in genes involved in the fatty acid β-oxidation pathway. The diagnosis of CoQ_10_ deficiencies is determined by the decrease of its content in skeletal muscle and/or dermal skin fibroblasts. Dietary CoQ_10_ supplementation is the only available treatment for these deficiencies that require a rapid and distinct diagnosis. Here we review methods for determining CoQ_10_ content by HPLC separation and identification using alternative approaches including electrochemical detection and mass spectrometry. Also, we review procedures to determine the CoQ_10_ biosynthesis rate using labeled precursors.

## 1. Introduction

The mitochondrial respiratory chain (MRC) generates most of the cellular ATP and is comprised of five multi-subunit enzyme complexes. Both the mitochondrial DNA (mtDNA) and the nuclear DNA (nDNA) encode for polypeptides of these complexes and also proteins involved in mitochondrial function. Besides MRC enzyme complexes, two electron carriers, coenzyme Q (CoQ) and cytochrome c, are vital for mitochondrial synthesis of ATP. Mutations in genes of either genome may cause mitochondrial diseases, which are common among inherited metabolic and neurological disorders [1].

CoQ is a lipid-soluble component of virtually all cell membranes. It is composed of a benzoquinone ring with a polyprenyl side chain, the number of isoprene units being a characteristic of given specie, e.g., 10 in humans (CoQ_10_). CoQ_10_ transports electrons from MRC Complexes I and II to Complex III. These electrons come from either NADH or succinate [2] although CoQ_10_ can be alternatively reduced with electrons provided by different redox reactions in mitochondria [3]. Consequently, CoQ_10_ is essential for ATP production inside mitochondria, although it is also an indispensible antioxidant in extramitochondrial membranes and a key factor for pyrimidine nucleotide synthesis [4].

CoQ biosynthesis depends on a pathway that involves at least 11 genes (*COQ* genes), showing a high degree of conservation among species, and is carried out by a putative multi-subunit enzyme complex [5]. Most of the information about the CoQ biosynthesis pathway comes from yeast, and maintains a high homology with mammal gene components (Table 1) [6]. The CoQ_10_ biosynthesis pathway is highly regulated by transcription factors PPARα and NFκB [7,8,9]. HuR and hnRNP C1/C2 binding proteins stabilize *COQ7* mRNA as another CoQ_10_ biosynthesis regulatory mechanism [10]. 

Coq7p is post-translationally regulated in yeast that involves mitochondrial phosphatase Ptc7 [11,12]. Ptc7 human orthologue (*PPTC7*) is related to cellular bioenergetics and stress resistance [13]. Coq7p activity is a key regulator of the CoQ biosynthesis complex [6,14], which may depend on the interaction with *Coq9p* contributing to the stabilization of the biosynthesis complex [15,16,17,18]. The level of CoQ is highly regulated inside cells and tissues but its concentration is different in each tissue and organ, and depends on dietary conditions and age [19,20]. CoQ also varies greatly in human diseases such as Alzheimer’s disease, cardiomyopathy, Niemann-Pick and diabetes.

## 2. CoQ_10_ Deficiency Syndrome

CoQ_10_ deficiency syndrome includes diverse inherited pathological diseases defined by the decrease of CoQ_10_ content in muscle and/or cultured skin fibroblasts. CoQ_10_ deficiency impairs oxidative phosphorylation and causes clinically heterogeneous mitochondrial diseases [21,22]. When the decrease in CoQ_10_ content is due to mutations in genes encoding proteins of the CoQ biosynthesis pathway or its regulation (COQ genes), it causes primary CoQ_10_ deficiency [23,24]. Secondary CoQ_10_ deficiencies may be due to defects in genes unrelated to the CoQ_10_ biosynthetic pathway. Secondary CoQ_10_ deficiency is a common finding in oxidative phosphorylation (OXPHOS) and non-OXPHOS disorders [25]. A low mitochondrial CoQ_10_ content is described in mtDNA depletion [26], mutations in the DNA repairing aprataxin [27], mutations of the enzyme *ETFDH* of the β-oxidation of fatty acids [28], recurrent food intolerance and allergies [29], methylmalonic aciduria [30], myalgic encephalomyelitis chronic fatigue syndrome [31], and propionic acidemia [32]. We propose that cases of secondary CoQ_10_ deficiency associated with OXPHOS defects could be adaptive mechanisms to maintain a balanced OXPHOS which is required to keep cells alive, although the mechanisms explaining these deficiencies and the pathophysiological role in the disease are unknown.

The clinical phenotypes of primary CoQ_10_-deficient patients are broader than initially reported in 1989 [33], including (i) a multisystem disorder with steroid-resistant nephrotic syndrome as the main clinical manifestation (*COQ1-PDSS2)* [34], (*COQ2*) [35], (*COQ6)* [36] and (*ADCK4*) [37]; (ii) a multisystem disorder without nephrotic syndrome (*COQ1*-*PDSS1)* [38], (*COQ9*) [39] and *(COQ7)* [40]; (iii) cerebellar ataxia (*COQ8*-*ADCK3)* [41,42,43,44,45,46,47]; and (iv) myopathy and encephalopathy (*COQ4*) [48,49,50]. 

## 3. Primary CoQ_10_ Deficiency Therapy

Primary CoQ_10_ deficiency is unique among mitochondrial diseases because an effective therapy is available for patients, which is the supplementation of CoQ_10_. Ubiquinol, the reduced form of CoQ_10_, was recently approved as an orphan drug for primary CoQ_10_ deficiency [51]

While this approach is quite successful in some patients, with a clear improvement of the pathological phenotype [52], some cases do not show any clinical relief as would be expected [53], probably because they are suffering secondary CoQ_10_ deficiency. High-dose oral CoQ_10_ supplementation can stop the progression of the encephalopathy and allows the recovery of renal damage [52]. High-dose CoQ_10_ supplementation was also able to prevent the onset of renal symptoms in *PDSS2*-deficient mice [54]. Furthermore, CoQ_10_ but not other quinones can restore mitochondrial function in deficient human fibroblasts [55]. Due to the therapeutic possibility of CoQ_10_ supplementation for these patients, a rapid and unequivocal diagnosis of the deficiency is essential. 

## 4. CoQ_10_ Determination in Cells and Tissues

Content of CoQ_10_ has been determined in plasma, white blood cells, skin fibroblasts and skeletal muscle biopsies to assess a deficiency diagnosis [56,57,58], and recently useful determination in the urine of pediatric patients was demonstrated [59]. Although CoQ can be measured in plasma and white blood cells, you cannot use it for the diagnosis of mitochondrial diseases since CoQ content in plasma and white blood cells is often not decreased in these conditions. 

CoQ_10_ content is mainly analyzed by the injection of lipid extracts in HPLC and detected by either electrochemical and/or UV-vis detectors, or mass spectrometry. Electrochemical detection has significant advantages compared to UV-vis detection; these include higher sensitivity and also the ability to measure oxidized and reduced forms of CoQ, either separately or combined, according to differential positioning of the conditioning cell (before or after the injector valve, respectively).

CoQ_10_ extraction from biological samples (0.5 mg protein) requires the disruption of hydrophobic elements (lipid bilayers and lipoproteins) by adding SDS (1% final concentration). Lipids are dispersed with an alcohol cocktail (2-propanol 5% in ethanol) mixed with the disrupted biological sample (ratio 1:2 *v*/*v*), and they undergo subsequent triplicated hexane extraction (dispersed sample:hexane ratio 3:5 *v*/*v*). Hexane fractions are mixed and dried under vacuum, and then reconstituted in ethanol prior to HPLC analysis. To estimate CoQ_10_ recovery, 100 pmol CoQ_9_ was included in the alcohol cocktail (2-propanol 5% in ethanol). Trace amounts of CoQ9 may have eventually been found in human tissues (probably from dietary uptake), but this does not interfere with the significant amount of internal standard added.

For convenience in high-throughput analysis, volumes are scaled down for extraction and vortex in 1.5 mL polypropylene tubes or 2 mL cryo vials.

Separation in C18 RP-HPLC columns (5 µm, 150 × 4.6 mm) requires 20 mM AcNH_4_ pH 4.4 in methanol (solvent A) and 20 mM AcNH_4_ pH 4.4 in propanol (solvent B). A gradient method with a 85:15 solvent mixture (A:B ratio), and a flow rate of 1.2 mL/min, is regularly used as the starting conditions. The mobile phase turns to a 50:50 A:B ratio starting in minute 6 and completed in minute 8, as the flow rate decreases to 1.1 mL/min. After 20 min (run time) at 40 °C, the columns are re-equilibrated to the initial conditions for three additional minutes.

**Figure 1:**
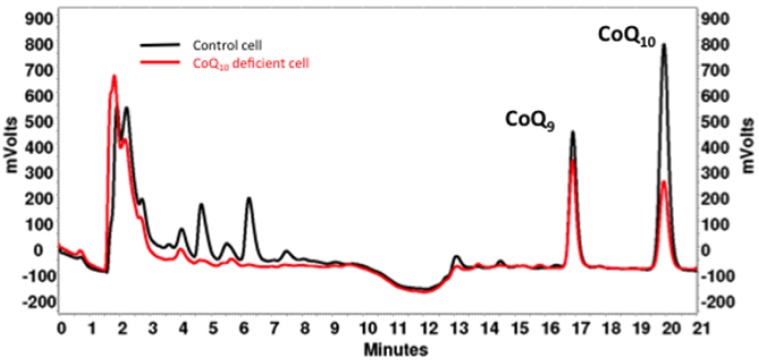
HPLC elution profile of lipid extracts from human skeletal muscular tissue. Patient pathological profile (red plot) shows that CoQ**_10_** is clearly diminished compared to healthy control volunteers (black plot). CoQ**_9_** is used as internal standard for normalization.

The detection of total CoQ_10_ can be achieved either by UV-vis (set to 275 nm) or electrochemical (ECD) detectors (channel 1 set to −700 mV and channel 2 set to +500 mV, conditioning guard cell after injection valve). For complex samples including many peaks, the CoQ_10_ peak is confirmed by spectral information (UV-vis) or by the redox area ratio (ECD detector, −700/+500 area ratio), compared to pure CoQ_10_. Figure 1 illustrates two chromatograms that correspond to normal age-matched human dermal fibroblasts (black plot) compared to patient dermal fibroblasts with CoQ_10_ deficiency (red plot).

## 5. Analysis of CoQ_10_ Biosynthesis

Another important approach to assess CoQ_10_ deficiency in cells is to determine the rate of biosynthesis by the level of incorporation of labeled of CoQ_10_ precursors such as *para*-hydroxybenzoate (*p*-HB) labeled with either ^13^C-*p*-HB or ^14^C-*p*-HB, which is the precursor of the benzoquinone ring, or ^2^H-mevalonate, which is the precursor of the isoprenyl side chain [10,60]. 

Polyprenyl-*p*HB transferase activity was assayed by measuring the incorporation of ^14^C-*p*-HB into nonaprenyl-4-hydroxybenzoate [35]. Isolated mitochondria (0.1–1 mg protein) were mixed with assay buffer (50 mM phosphate buffer, pH 7.5, 10 mM MgCl_2_, 5 mM EGTA containing 1 mM PMSF, 20 μg/mL each of the protease inhibitors chymostatin, leupeptin, antipain, and pepstatin A, 5 μM solanesyl pyrophosphate solubilized in detergent solution (1% in water), and 10^5^ DPM of ^14^C-*p*-HB). A sufficient volume of a 10% detergent stock solution was also added to the reaction medium to achieve a final detergent concentration of 1%. The following detergents were tested: Triton X-100, Chaps, sodium cholate, sodium deoxycholate, lysophosphatidyl choline, and octylglucoside. After incubation for 30 min at 37 °C with gentle stirring, the reaction was stopped by chilling samples to 4 °C. Prenylated ^14^C-*p*-HB was separated by organic extraction with hexane and then measured using a liquid scintillation counter. Specific activity was expressed as disintegrations per minute (DPM) min^−1^·mg·protein^−1^.

**Figure 2:**
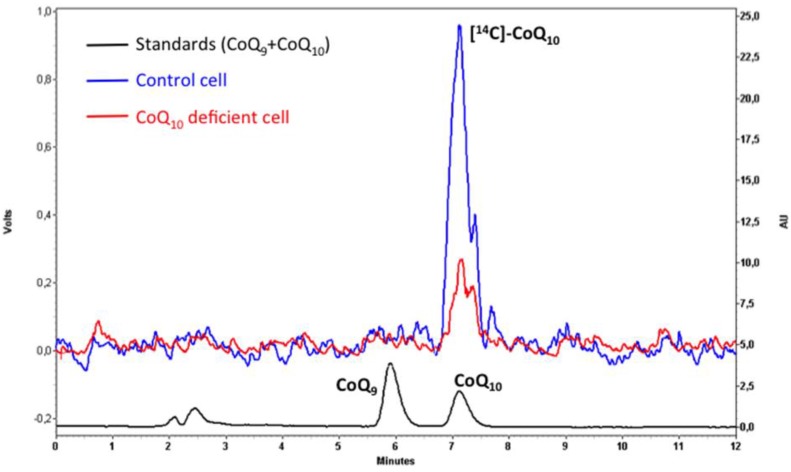
HPLC elution profile of lipid extracts from human fibroblasts cultured with the radiolabeled precursor ^14^C-*p-*HB. Patient pathological profile (red plot) shows that CoQ**_10_** is clearly diminished compared to control cells from healthy humans (blue plot). Left Y-axis shows the radio-flow detector scale (volts). Right Y-axis shows the UV-detector scale (absorbance units) for a standard pool of CoQ**_10_** and CoQ**_9_** (black plot). Notice that the only peak detected in this analysis corresponded with CoQ**_10_**.

Biosynthesis of ^14^C-CoQ_10_ has been quantified in any type of cell culture, such as cancer cells, human skin fibroblasts, and murine embryonic fibroblast and stem cells [10,61]. Previously, cultures were incubated with 4.5 nM ^14^C-*p-*HB for one to three days, depending on the cell-specific rate of growth. The ^14^C-*p-*HB was chemically synthesized in our laboratory from ^14^C-thyrosine [61]. Labeled-CoQ_10_ content is analyzed by lipid extract injection in HPLC and detected by the radio-flow detector LB 509 with a solid cell YG 150 Al-U4D (Berthold Technologies, Bad Wildbad, Germany) in parallel with either electrochemical or UV-vis detectors. Lipid extraction is done as we described above for CoQ_10_ determination in cells and tissues, but isocratic HPLC analysis lipid separation is performed with methanol:propanol (65:35) plus 20 mM AcNH_4_ pH 4.4 at a constant flow rate of 1 mL/min (Figure 2).

Alternatively, a non-radioactive protocol to analyze CoQ_10_ biosynthesis was developed using either ^2^H-mevalonate or ^13^C-phydroxybenzoate as CoQ_10_ precursors as described by Buján et al. (2014) [60]. Human fibroblasts at 60%–70% were incubated with these precursors for 24–72 h at different concentrations. After incubation, cells were trypsinized and washed twice with isotonic buffer. Pelleted cells were resuspended with 300 μL of a buffer solution containing 0.25 mmol/L sucrose, 2 mmol/L EDTA, 10 mmol/L Tris and 100 UI/mL heparin, pH 7.4, and sonicated twice for 5 s. These homogenates were used to determine CoQ_10_ biosynthesis measuring by HPLC-MS/MS, as described in Arias et al. (2012) [62]. Briefly, HPLC separation was as indicated above and extracted peaks were analyzed by MS/MS in a Micromass Quattro micro™ (Waters/Micromass, Manchester, UK). The MS/MS was operated in the electrospray positive ion mode with a cone voltage (CV), and collision energy (CE) of 15 V and 20 eV, respectively. The following multiple-reaction monitoring transitions were selected: *m*/*z* 900 > 203 and 897>197 for ^13^C-CoQ_10_ or ^2^H-CoQ_10_, respectively, 894 > 197 for the physiological CoQ_10_ and 826 > 197 for CoQ_9_ (internal standard). The dwell time for each transition was 200 ms and the run-time was 16 min. Nitrogen (at a flow rate of 50 L/h) and argon (adjusted to obtain a vacuum of 3°—10^−3^ bar) were used as the nebulizing and collision gas, respectively.

## 6. Concluding Remarks

Coenzyme Q_10_ deficiency syndrome includes a group of mitochondrial diseases showing diverse inherited pathological phenotypes. The common aspect of them is the lower content of CoQ_10_ in tissues and organs. Primary deficiency is caused by defects in proteins encoded by *COQ* genes, which are components of the biosynthesis pathway or its regulation. CoQ_10_ supplementation is the current treatment of primary CoQ_10_ deficiency, which highly improves symptoms. A rapid and distinct characterization of the deficiency is important, and it is mainly determined in skeletal muscle and/or skin dermal fibroblasts. The main approach is to analyze the total content of CoQ_10_ in lipid extracts by HPLC and UV and/or electrochemical detection. Alternatively, the CoQ_10_ biosynthesis rate in cultured cells can be determined by incubation with radiolabeled precursors.

## Figures and Tables

**Table 1:** Yeast *COQ* genes and their characterized human homologues.

Yeast	Human	Function
*COQ1*	PDSS1 */PDSS2 *	Synthesis of polyprenyl-diphosphate
*COQ2*	COQ2 *	*p*HB-prenyl-transferase
*COQ3*	COQ3 *	Methyltransferase
*COQ4*	COQ4 *	Organization of the multi-enzyme complex
*COQ5*	*COQ5*	Methyltransferase
*COQ6*	COQ6 *	Mono-oxygenase
*COQ7*	COQ7 *	Hydroxylase
*COQ8*	ADCK3 */ADCK4 *	Unorthodox kinase (regulatory)
*COQ9*	COQ9 *	Lipid binding protein
*COQ10*	*COQ10A/COQ10B*	CoQ chaperone
*PTC7*	*PPTC7*	Phosphatase (regulatory)

* These genes were mutated in human causing primary CoQ_10_ deficiency.
